# A scoping review on e-cigarette environmental impacts

**DOI:** 10.18332/tpc/172079

**Published:** 2023-10-02

**Authors:** Gabrielle Ngambo, Elizabeth G. Hanna, John Gannon, Hannah Marcus, Marta Lomazzi, Razieh Azari

**Affiliations:** 1 Department of Social and Preventive Medicine, Faculty of Medicine, Université Laval, Quebec, Canada; 2 Fenner School of Environment and Society, Australian National University, Canberra, Australia; 3 World Federation of Public Health Associations, Geneva, Switzerland; 4 Institute of Global Health, Faculty of Medicine, University of Geneva, Geneva, Switzerland; 5 Faculty of Translation and Interpreting, University of Geneva, Geneva, Switzerland

**Keywords:** e-cigarette, tobacco, environment, environmental impact, scoping review

## Abstract

**INTRODUCTION:**

The use of e-cigarettes has grown in popularity worldwide. From their manufacturing, use, and disposal, the environmental impacts of e-cigarettes present a novel public health concern that needs to be urgently investigated. However, very limited studies have focused on the subject matter. The present study aims to review available studies to identify the environmental impacts of e-cigarettes.

**METHODS:**

In this scoping review, we undertook a search in two databases (PubMed and Web of Science) from inception until 21 March 2023, and a gray literature search in Google Scholar. Reference lists of publications included in the scoping review were screened manually for additional relevant publications. Scientific publications that were in English and focused on the potential impacts of e-cigarettes on the environment were included.

**RESULTS:**

A total of 693 publications were identified, of which 33 were subjected to full-text review and 9 publications were finally included in the review. The impacts on air quality, water, land use, and animals, water and energy consumption, with associated environmental impacts, increased pollution and emissions due to greater e-cigarette production, having harmful and toxic components, creating pollution and waste issues, and global environmental impacts due to manufacturing and importing ingredients and components from low- and middle-income countries, were identified as the environmental impacts of e-cigarettes.

**CONCLUSIONS:**

Despite the emphasis on the environmental threat of e-cigarettes, there are limited scientific studies on the environmental impacts of the e-cigarette life cycle. Considering the rapid expansion of e-cigarette usage, there is an urgent need for a rigorous assessment of their life-cycle environmental burden of the various potential health, environmental, and other consequences.

## INTRODUCTION

Consumption and sales of electronic cigarettes (e-cigarettes) have risen dramatically worldwide^1,2^. Sixty million e-cigarettes and refills are sold annually, and one-third of these are designed for single-use in the United States^3^. The global market value was estimated to grow from US$ 14.53 billion in 2017 to US$ 48.9 billion by 2025^4^. E-cigarettes are highly popular among youth and young adults^1,3^. Contradictory and confusing information exists concerning public health risks and benefits of e-cigarettes^2^. For example, their growing popularity can partly be attributed to e-cigarettes being marketed to the public as ‘healthier alternatives’ and ‘eco-friendly’ compared to conventional cigarettes^2,3^. However, several scientific studies suggest that e-cigarettes may have short- and long-term health effects^2^.

E-cigarettes are the most common electronic nicotine delivery systems (ENDS)^1,2^. These electronic battery-powered devices heat liquid (e-liquid) generally containing nicotine and flavoring agents to become aerosols inhaled by the user^1-3^. Some e-cigarettes are reusable, which means that batteries, capsules, and atomizers can be replaced, while others are only single-use^1^. Although e-cigarettes provide users with the sensation of smoking known as vaping, they are promoted as being tobacco-free and do not require burning for consumption^2,3^.

Although information exists about the direct impact of e-cigarettes on health, very little scientific evidence exists concerning the environmental impact of the life cycle of these products and their potential indirect health harm^1-9^. From their manufacturing processes to their use and disposal, the environmental impact of e-cigarettes presents a novel public health concern that needs to be urgently investigated^5^. An example is that e-cigarettes are a growing waste management concern because, despite their small size, they are consumed and discarded much more quickly than typical electronics^1-9^.

General e-waste can contain precious metals like gold, copper, and nickel, as well as rare materials like indium and palladium^6,7^. According to the Basel, Rotterdam, and Stockholm Conventions, the presence of toxic materials, such as mercury, lead, or brominated flame retardants, justifies classifying e-waste as ‘hazardous’^6,7^. Furthermore, e-waste is the fastest-growing hazardous waste stream^8,9^. In 2019, 53.2 million metric tons (Mt) of e-waste was generated worldwide and is expected to reach 74.7 million Mt by 2030^8,9^.

Tobacco companies, including e-cigarette industries, recognize that they need to address novel environmental impacts caused by their growing use of batteries and other electronics in e-cigarettes^5^. Yet, despite recognition of the potential hazards, eco-friendly claims are often used as a marketing strategy by the tobacco industry^5,10^. If these claims are shown to be false, then ‘greenwashing’ needs to be called out to avoid misinformation being used as a tool to unethically drive consumer demand. Hence, there is a need to evaluate the environmental impacts of e-cigarettes and to expand the publicly accessible literature documenting such evidence^11^. For these reasons, a scoping review was conducted to systematically review available studies to identify the environmental impacts of e-cigarettes. Underlying this approach was the fundamental aim of promoting expanded knowledge and awareness on this overlooked issue, so that comprehensive response policies and strategies may be initiated to mitigate observed and projected health and environmental consequences.

## METHODS

This scoping review of peer-reviewed and grey literature was prepared according to the framework of the Preferred Reporting Items for Systematic Review and Meta-analysis (PRISMA)^12^ guidelines.

Two databases including PubMed and Web of Science were searched from inception to 21 March 2023 to identify relevant literature. Grey literature was searched using Google Scholar and the first 100 search results sorted by relevance were compared against the inclusion criteria. Moreover, reference lists of publications included in the scoping review were screened manually for additional relevant publications. At this stage, no language restriction was applied.

The search for the relevant literature was conducted using the following keywords in the title and abstract of the literature: e-cigarettes, vaping, climate, environment, planet, and waste. The final search results were exported into an Excel spreadsheet, and duplicates were removed.

Publications were included if they satisfied the following eligibility criteria: 1) all types of scientific publications such as articles, editorials, viewpoints, guidelines, etc.; and 2) English-language publications that focused on the potential impacts of e-cigarettes on the environment.

**Figure 1:**
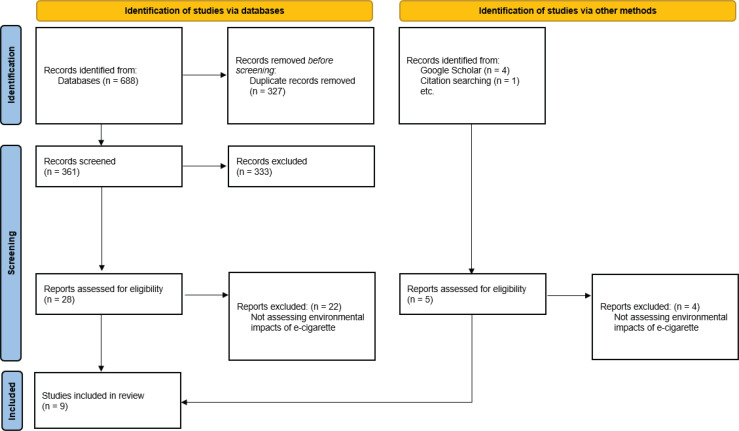
PRISMA flow diagram of the scoping review process

The selection of relevant publications was conducted in three stages: 1) screening of the title and abstract conducted by two authors; 2) full-text screening completed independently by two authors and raised discrepancies resolved through discussion until consensus was reached; and 3) data extraction and collation. These stages are summarized in the PRISMA flow diagram (Figure 1).

Eligible publications were reviewed by two authors independently and relevant details were extracted which related to the potential impact of e-cigarettes on the environment: namely their components, their chemical contaminants, their waste issues, and pollution (Figure 1). Discrepancies were resolved through discussion until a consensus was reached.

## RESULTS

A total of 693 publications were identified by the search (of the above-mentioned databases and additional publications through manual search). Duplicated records (n=327) were removed. Based on the eligibility criteria, the titles and abstracts of 361 publications were assessed for their relevance, resulting in 33 publications being retained. The full texts of the remaining 33 publications were obtained, and after applying the eligibility criteria, a total of 9 publications met the criteria and were included in this scoping review (Figure 1)^1^,^2,3,5,11,13-16^. Characteristics of the included publications are shown in Table 1.

**Table 1:** Characteristics of nine studies, included in the scoping review from inception until 21 March 2023, reporting the environmental impacts of e-cigarettes

*Authors*	*Year*	*Methods*	*Assessment*	*Conclusions*
Chang^11^	2014	Systematic review	Gaps in research related to the environmental impact of e-cigarettes	Little is known about the environmental impact of e-cigarettes life cycle
Krause and Townsend^14^	2015	Quantitative	Testing e-cigarettes with Toxicity Characteristic Leaching Procedure (TCLP) and California Waste Extraction Test (WET) to assess the potential of e-cigarettes to be a hazardous waste in the USA	Some e-cigarettes should be classified as hazardous waste for lead leaching
Lerner et al.^16^	2015	Semi-quantitative	Testing the presence of oxidants/ROS in ENDS products (e-cigarettes)	Data on dichloro- fluorescein (DCF) is indicative of oxidant presence but not an accurate measurement of ROS levels
Lee et al.^13^	2017	Quantitative	Assess the content of e-cigarettes emissions using e-cigarette aerosol generation, sampling system, chromatographic and spectroscopic methods	E-cigarettes contain some pollutants
Hendlin^5^	2018	N/A	Information on e-waste	N/A
Marcham and Springston^2^	2019	Review of the literature	E-cigarettes in the indoor environment	E-cigarettes should be considered a source of aerosols, VOCs, and particulates in the indoor environment
Papaefstathiou et al.^3^	2019	Review of the literature	Main and side effects of e-cigarettes	E-cigarettes are non-combustible products, and when heated, they produce aerosols that might be toxic
Beutel et al.^1^	2021	Review of the literature	A review of the environmental pollution of e-cigarettes and cigarettes	E-waste may be a pollutant source
Pourchez et al.^15^	2022	N/A	E-cigarettes as a new environmental threat	N/A

All publications included were published between 2014 and 2022. Four publications presented reviews of the literature^1-3,11^, 2 publications quantitative^13,14^, and 1 publication semi-quantitative methods^16^ (Table 1).

The publications under review aimed to: assess the research gaps related to the environmental impacts of e-cigarettes due to their manufacture, use and disposal^11^, assess the potential of e-cigarettes to be classified as hazardous waste^14^, test the presence of oxidants/reactive oxygen species (ROS) in ENDS products^16^, assess the content of e-cigarette emissions^13^, provide information on e-cigarette disposal^5^, investigate the potential exposures and risks from the use of e-cigarettes, particularly for bystanders, in the indoor environment^2^, investigate the potential health, safety and environmental effects of e-cigarettes^3^, discuss the cigarette and e-cigarette contamination in the context of environmental sources and impacts^1^, and discuss e-cigarettes as a new environmental threat^15^ (Table 1).

All the publications under review stated that studies on the environmental impact of e-cigarettes are limited. However, all the publications shared one common concern, which is the environmental threat of e-cigarettes. Limited scientific information on the environmental impacts of e-cigarette life cycle^11^, classifying some e-cigarettes as hazardous waste for lead leaching^14^, raising concerns regarding the safety of e-cigarettes use and the disposal of e-cigarette waste products into the environment due to the presence of oxidants/ROS, nanoparticles, and copper metals associated with the e-cigarette aerosols intended for inhalation^16^, the presence of toxic compounds including nicotine, fine and nanoparticles, carbonyls, and some toxic volatile organic compounds (VOCs) such as benzene and toluene in e-cigarettes aerosols^13^, considering e-cigarettes as a source of aerosols, VOCs, and particulates in the indoor environment^2^, considering the fact that e-cigarettes are non-combustible products, and when heated, they produce aerosols that might be toxic^3^, and considering e-cigarette use and disposal as a pollutant source^1^, were stated as the main outcomes of the publications (Table 1).

All the publications under review reported several reasons and potential impacts of e-cigarettes on the environment, which can be categorized into three main areas: production, use, and disposal. The impacts on air quality, water, land use, and animals, due to the production, use and disposal of e-cigarettes^1-3,5,11,13-15^, water and energy usage, with associated environmental impacts, required for manufacturing e-cigarette components^1,2,5,11,14,16^, increased pollution and emissions due to greater e-cigarette production^11^, having harmful components like e-liquid (e.g. nicotine), batteries, toxic chemical (e.g. VOC, PM, lead)^1,5,11,13,15^, creating pollution and waste issues^1-3,5,11,13-15^, and global environmental impacts due to manufacturing and importing ingredients and components of e-cigarettes from low- and middle-income countries^5,11^, were reported by the publications under review as the environmental impacts of e-cigarettes (Table 2).

**Table 2:** Potential impacts of e-cigarettes on the environment reported by the publications under the scoping review from inception until 21 March 2023

*Impacts*	*Chang^11^*	*Krause and Townsend^14^*	*Lerner et al.^16^*	*Lee et al.^13^*	*Hendlin^15^*	*Marcham and Springston^2^*	*Papaefstathiou et al.^3^*	*Beutel et al.^1^*	*Pourchez et al.^15^*
Impacts on the air, water, land use quality, animals	x	x		x	x	x	x	x	x
Production of e-cigarette components requires energy and water	x	x	x		x	x		x	
Larger factories of e-cigarettes generate greater emissions in the environment	x								
E-cigarettes contain harmful components like e-liquid (e.g. nicotine), batteries, toxic chemical (e.g. VOC, PM, lead)	x				x		x	x	x
E-cigarettes create pollution and waste issues	x	x		x	x	x	x	x	x
Global environmental impacts because e-cigarette components and ingredients are imported, and wastes are shipped to developing countries	x				x				

## DISCUSSION

This study selectively reviewed the available literature to identify the environmental impacts of e-cigarettes. The results of the present study indicate that air quality, water, land use, and animals, are threatened due to the production, use, and disposal of e-cigarettes^1-3,5,11,13-15^. Still, evidence remains limited regarding the environmental impacts of e-cigarettes^1-3,5,11,13-16^. Furthermore, no studies have formally evaluated the environmental impacts of the life cycle of e-cigarettes^1,9^. It is also unclear how the environmental impacts of e-cigarettes compare to those of conventional cigarettes^11^. For instance, it could be informative to compare the life cycle pollution from production to disposal between traditional cigarettes and e-cigarettes. Despite the absence of supporting data or environmental impact studies, eco-friendly claims have been used by manufacturers as a marketing strategy to promote e-cigarettes to consumers^3,11^.

### Production

In regard to production, it is worth noting that e-cigarettes contain a battery, a heating element, an atomizer (aerosolization chamber), a cartridge, an e-liquid, and a mouthpiece^2,5,11,14,16^. Manufacturing the product is an energy-consuming process with associated environmental impacts^1^,^2,5,11,14,16^. For example, extraction and purification of nicotine from the tobacco plant requires a large amount of water and generates non-recyclable halogenated waste and pollution^1,2,5^. Also, as a result of e-cigarette marketing, the demand for tobacco crops could potentially increase, which would present a potential alteration in land use^11^. Greater e-cigarette production demand drives increased pollution (e.g. greenhouse gas emissions), therefore contributing to processes that may lead to climate change^11^. Due to a lack of regulations in countries like the US, data on pollutant contamination of water, land, and air may not be obtained from manufacturing sites^11^. However, global environmental impacts are important to consider because ingredients and components of e-cigarettes are manufactured and imported from low- and middle-income countries including India^5,11^.

### Use

In addition to environmental harms from production, understanding the potential environmental impact of the use of e-cigarettes is important. Beside the direct harm experienced by users, e-cigarette vapors are potent sources of air pollution such as aldehydes, carbon monoxide, particulate matter (PM), VOCs, heavy metals, and nicotine^1,3,13^. Compared to smoke from conventional cigarettes, the amount of PM and heavy metal emissions from e-cigarette vapor were found to be similar or greater^2,13^. The potential risks of passive e-cigarette exposure among bystanders needs to be considered^2^. Because e-cigarette emissions contain measurable amounts of toxic chemicals, further investigation should be conducted to better understand the full scope of the environmental implications^2,13^.

### Disposal

The disposal of e-cigarettes should also be considered when studying their environmental impacts. E-waste is a major environmental problem, with a 2017 estimate suggesting that up to 45 billion kg of e-waste are discarded annually^5^. Increased use of e-cigarettes has led to a rise in the release of e-cigarette waste and related contaminants into the environment^1^.

Some e-cigarettes are designed to be completely disposable, while others are rechargeable^2,14^. Disposable e-cigarettes and vaping pods, spent e-cigarette capsules or replaceable pods, pose the most significant potential environmental burden^5,15^. Vaping pods are an example of plastic waste because they are not biodegradable and are poorly recyclable^15^. Also, they contain similar waste components as reusable e-cigarettes but are used for a shorter time before being discarded^5^.

Components like batteries and replaceable capsules containing concentrated nicotine residues can leach pollutants into water, air, and soil^1^. A particularly serious threat of environmental pollution is the littering of e-liquid containers^1^. They may contain high concentrations of residual nicotine, of known and unknown toxicity, and flavoring additives such as aldehydes^1,3^.

Therefore, e-cigarettes have different types of waste, including biohazard, plastic, and electronic waste^15^. We contend that the potential waste load from e-cigarettes exceeds that of traditional cigarettes due to the larger amount of components^3^. E-cigarette components like nicotine, lithiumion batteries, and electronic circuit boards, are considerable forms of biohazard and electronic waste^1,3,5,13-16^. On the one hand, the biohazard waste (nicotine, lithium-ion batteries) risk arises when e-cigarettes are improperly discarded and when broken components leach heavy metals (e.g. mercury, lead) and release toxic chemicals into the environment, affecting humans and animals^1,3,5,13-16^. These products can then bioaccumulate in animals and humans, creating health issues^1,3,5,8-11^. On the other hand, the electronic waste risk occurs when discarded components like batteries pose an explosion and fire hazard in waste and recycling facilities^15^. Biohazard and electronic waste should not be discarded in regular trash and instead should be disposed of in specific facilities^5,14,15^. However, regulation of such requires strengthened legislation and policies to be enacted. The likelihood of universal compliance with such regulation presents an additional and significant challenge for claims of e-cigarettes’ environmental safety.

The Basel, Rotterdam, and Stockholm conventions are science-based, legally binding global treaties aimed at protecting human health and the environment from hazardous chemicals and wastes. However, non-compliance is common as the global transport of waste continues to expand. Most e-waste from Western countries is shipped to developing countries, shifting the dangers and pollution-related risks to settings that are often least able to adequately address and mitigate them5. This rich-to-poor country shift transfers risk and harm whilst further exacerbating global health inequities. Moving forward, e-cigarettes will need to be part of the ongoing conversation on how to better govern and enforce such regulations under the broader aim of reducing the global environmental injustices that practices such as offshoring pollution continue to contribute towards.

### Strengths and limitations

The present study’s strength is that it is the first scoping review conducted to identify the environmental impacts of e-cigarettes. However, the limitations of our study need to be considered. One limitation of our study is that only two databases were searched, which might have led to the absence of relevant studies. More databases can be included for future studies. Manual screening of reference lists of publications included in the scoping review was used to add relevant publications that had not been initially identified through database searching.

Although it ensured that the review was exhaustive, some conclusions may have been influenced by this manual search strategy. Despite not restricting the language of publication, only English keywords were searched, which may have led to the exclusion of non-English publications. Another limitation is that only publications written in English were included. Future reviews should include non-English studies to have a better understanding of the situation.

## CONCLUSIONS

E-cigarette emissions and waste contain measurable amounts of nicotine and other toxic chemicals, thereby serving as significant sources of environmental pollution. Despite the emphasis on the environmental threat of e-cigarettes, there are limited scientific studies on the environmental impacts of the e-cigarette life cycle (manufacturing, use, and disposal). This life cycle is not studied enough for its impacts on human health associated with environmental pollution. As a result, critical ecosystems providing clean water, air, and food production, can be negatively affected. Although limited data have been reported about the life cycle of e-cigarettes, they may represent a significant long-term environmental threat due to the toxic nature of their composition. E-cigarette environmental impacts can be prevented with improved regulation of their production, use, and disposal. For example, the gradual elimination of disposable e-cigarettes in favor of reusable e-cigarettes and proper recycling and waste management could reduce environmental damage. It is important to note that ‘absence of evidence is not evidence of absence’. Since e-cigarettes are mainly owned by the tobacco industry, it is important to question whether vaping is more eco-friendly than smoking, as companies claim. Given the rapid expansion of e-cigarette manufacture, distribution, use, and disposal globally, a rigorous assessment of their life-cycle environmental burden of the various potential health, environmental, and other consequences is urgently required.

## Data Availability

The data supporting this research are available from the authors on reasonable request.
